# Isochaihulactone-induced DDIT3 causes ER stress-PERK independent apoptosis in glioblastoma multiforme cells

**DOI:** 10.18632/oncotarget.13266

**Published:** 2016-11-10

**Authors:** Sheng-Feng Tsai, Michael Tao, Li-Ing Ho, Tzyy-Wen Chiou, Shinn-Zong Lin, Hong-Lin Su, Horng-Jyh Harn

**Affiliations:** ^1^ Department of Life Sciences, National Chung-Hsing University, Taichung, Taiwan; ^2^ Department of Bioengineering, University of California, San Diego, La Jolla, California, USA; ^3^ Department of Chest Medicine, Taipei Veterans General Hospital, Taipei, Taiwan, School of Medicine, National Yang-Ming University, Taipei, Taiwan; ^4^ Department of Life Science, Graduate Institute of Biotechnology, National Dong Hwa University, Hualien, Taiwan; ^5^ Bioinnovation Center, Tzu Chi foundation, Department of Neurosurgery, Hualien Tzu Chi Hospital, Hualien, Taiwan; ^6^ Bioinnovation Center, Tzu Chi Foundation, Department of Pathology, Buddhist Tzu Chi General Hospital, Hualien, Taiwan; ^7^ Department of Pathology, China Medical University Hospital, Taichung, Taiwan

**Keywords:** ER stress, glioblastoma, DDIT3, isochaihulactone, NAG-1

## Abstract

The endoplasmic reticulum (ER) is a major site of cellular homeostasis regulation. Under the ER stress condition, Glioblastoma multiform (GBM) cells activate the unfolded protein response. In this study, we discovered isochaihulactone, a natural compound extracted from the Chinese traditional herb Nan-Chai-Hu, which can disrupt ER homeostasis in GBM cell lines. It can induce DNA damage inducible transcript 3 (DDIT3) expression which is independent of 78 kDa glucose-regulated protein (GRP78) and protein kinase RNA-like endoplasmic reticulum kinase (PERK) expression. Flow cytometry results revealed that isochaihulactone trigger the cell cycle arrest at G2/M phase and apoptosis in GBM cells. Isochaihulactone induced DDIT3 led to the expression of NAG-1. The *in vivo* study showed that isochaihulactone suppressed tumor growth, and DDIT3 and Caspase3 overexpressed in the xenograft model, which is consistent with the *in vitro* study. Overall, the data revealed that isochaihulactone disrupted ER homeostasis in cancer cells by increasing DDIT3 and NAG-1 expression. Our finding also provides a therapeutic strategy by using isochaihulactone for GBM treatment.

## INTRODUCTION

The endoplasmic reticulum takes part in several cellular functions, including protein synthesis, calcium homeostasis, and phospholipid synthesis [1]. ER stress is always present in cells undergoing rapid proliferation, hypoxia, and therapeutic treatment [2]. During ER stress, increased levels of unfolded proteins accumulate and cause protein kinase RNA-like endoplasmic reticulum kinase (PERK), an ER stress sensor, to activate. Moreover, the other ER stress sensor, inositol-requiring enzyme 1α (IRE1α), also activates the translation of HSP90 chaperone family member GRP78. GRP78 is involved in the subsequent folding steps for a subset of ER client proteins, a process called the unfolded protein response (UPR) [3]. In tumor cells, ER stress may restore homeostasis and render the adjacent environment hospitable for tumor survival and growth [4]. GRP78, are highly expressed in tumor cells, and contribute to cancer progression [5]. The UPR is usually inactive in normal cells, but is widely overexpressed in many cancers, including breast cancer [6], leukemia [7], colon cancer [8], and brain cancer.

Glioblastoma multiform and anaplastic astrocytoma, the most common and severe primary gliomas in adults, are highly mobile, invasive, and difficult to resect completely through surgery. PERK, DDIT3 and GRP78 are all expressed at low levels in the normal adult brain, but GRP78 are highly express in malignant GBM cell lines [9], and in GBM patients [10]. With increased GRP78 expression, this will lead to poor survival rate. In recent years, researchers have begun studying the potential role of GRP78 in GBM therapy [11–13]. Tian *et al*. used a different strategy in which increased levels of ER stress activated DDIT3 expression directly which led to cell apoptosis [14].

Nan-Chai-Hu (Chai Hu of the South), the root of Bupleurum scorzonerifolium, is a valuable Chinese herb used in the treatment of influenza, fever, malaria, cancer, and menstrual disorders in China, Japan, and many other parts of Asia. Previous studies have reported that the compound isochaihulactone (K8), isolated from the crude acetone extract of *B*. scorzonerifolium, causes cell cycle arrest at G2/M phase in lung adenocarcinoma A549 cells, and activates non-steroidal anti-inflammatory drug-activated gene (NAG-1) expression, thereby triggering A549 cell apoptosis [15]. By using a microarray, we discovered K8 induced DDIT3. However, the relationship between DDIT3 and NAG-1 has not been determined for K8 against GBM.

In this study, we used K8 to treat GBM cell lines, and discovered that GRP78 was not activated and PERK was downregulated under K8 treatment, despite strong DDIT3 expression. Our results proved that K8 upregulate DDIT3 expression, but will not activate PERK; previous studies have proven that PERK modulates DDIT3 expression. We subsequently demonstrated that DDIT3 increased NAG-1 expression in GBM cell lines, which led to tumor cell apoptosis. Finally, K8 reduced the tumor size considerably, and increased DDIT3 and caspase-3 expression *in vivo*.

## RESULTS

### ER-Stress-related proteins are widely expressed in glioblastoma multiform cell lines

**Figure 1:**
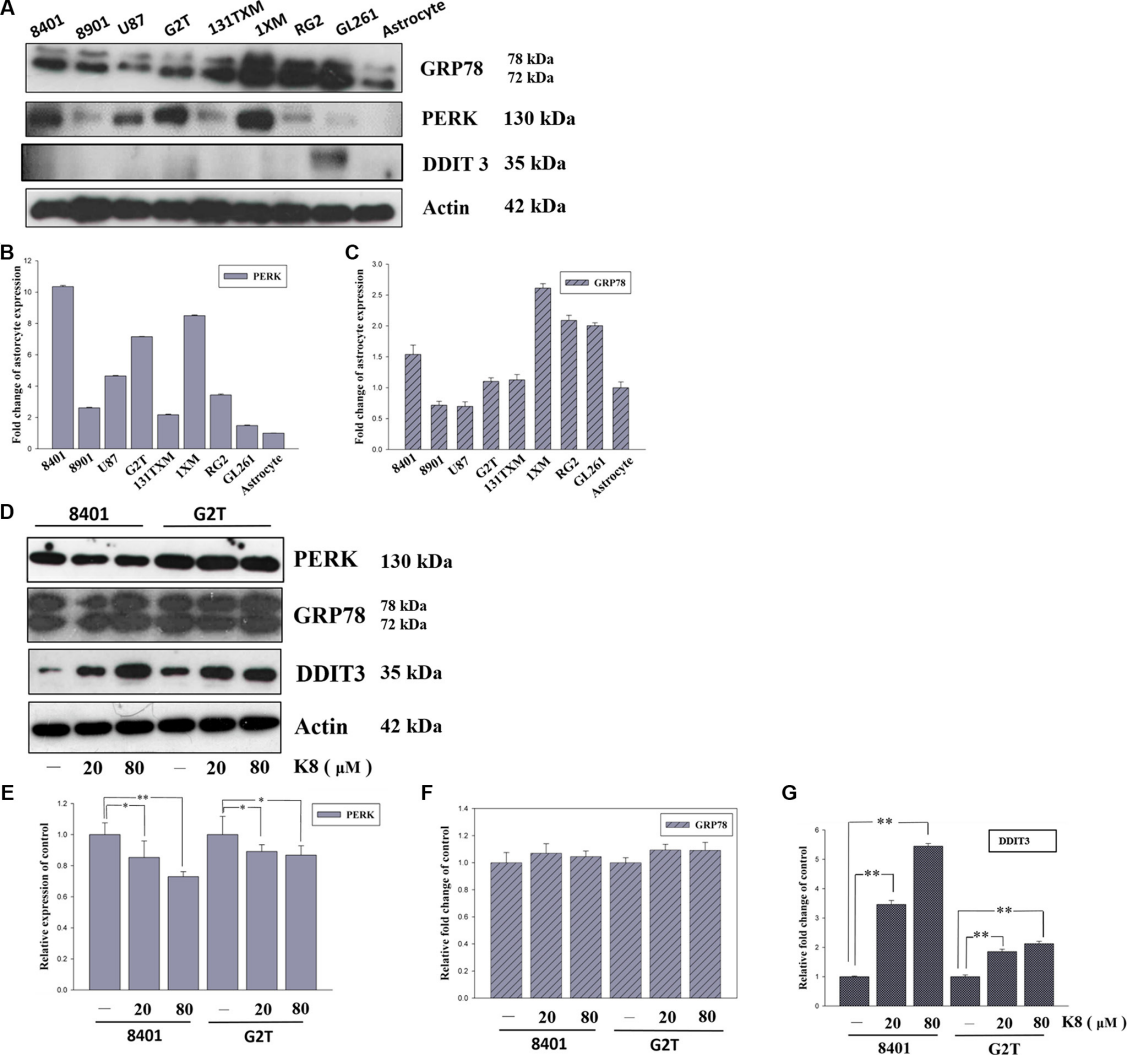
K8 induced DDIT3 expression in glioblastoma cell lines (**A**) ER stress marker expression basal level in glioblastoma cell line. (**B**–**C**) Quantification of PERK and GRP78 expression basal level compared with astrocyte. (**D**) Protein expression of ER stress markers were performed by Western blotting within K8 treatment in 24 hours. (**E**–**G**) Quantification showed PERK expression downregulated, GRP78 was not affected, and there was significant increase of DDIT3 compared to the control group. Values shown are the mean ± S.E.M. from three experiments. Level of statistical significance: *p < 0.05, **p < 0.01.

The ER-related proteomic profile was used to compare the ER stress level in different cell lines. Each GBM cell line, 8401, 8901, U87, G2T, 131TXM, 1XM, RG2, and GL261, showed high levels of ER stress marker PERK and UPR major modulator GRP78 (Figure 1A), compared with human primary astrocyte. These levels exhibited no correlation with respect to ER stress apoptosis-related protein DDIT3 (Figure 1A). As shown in Figure 1B, quantified data revealed there are two cell lines, 8401 and G2T, with high level PERK expression but lower level of downstream protein DDIT3. Therefore, we suggest that DDIT3 expression might not depend on PERK activation. Figure 1D shows the DDIT3, PERK, and GRP78 expression profiles in 8401 and G2T, following K8 treatment. We discovered that the level of the ER stress modulator GRP78 did not change following K8 treatment (Figure 1F). However, with K8 treatment, PERK expression was down regulated (Figure 1E). DDIT3, was consequently strongly expressed (Figure 1G).

### K8 is a potential drug that can induce glioblastoma cell death

**Figure 2:**
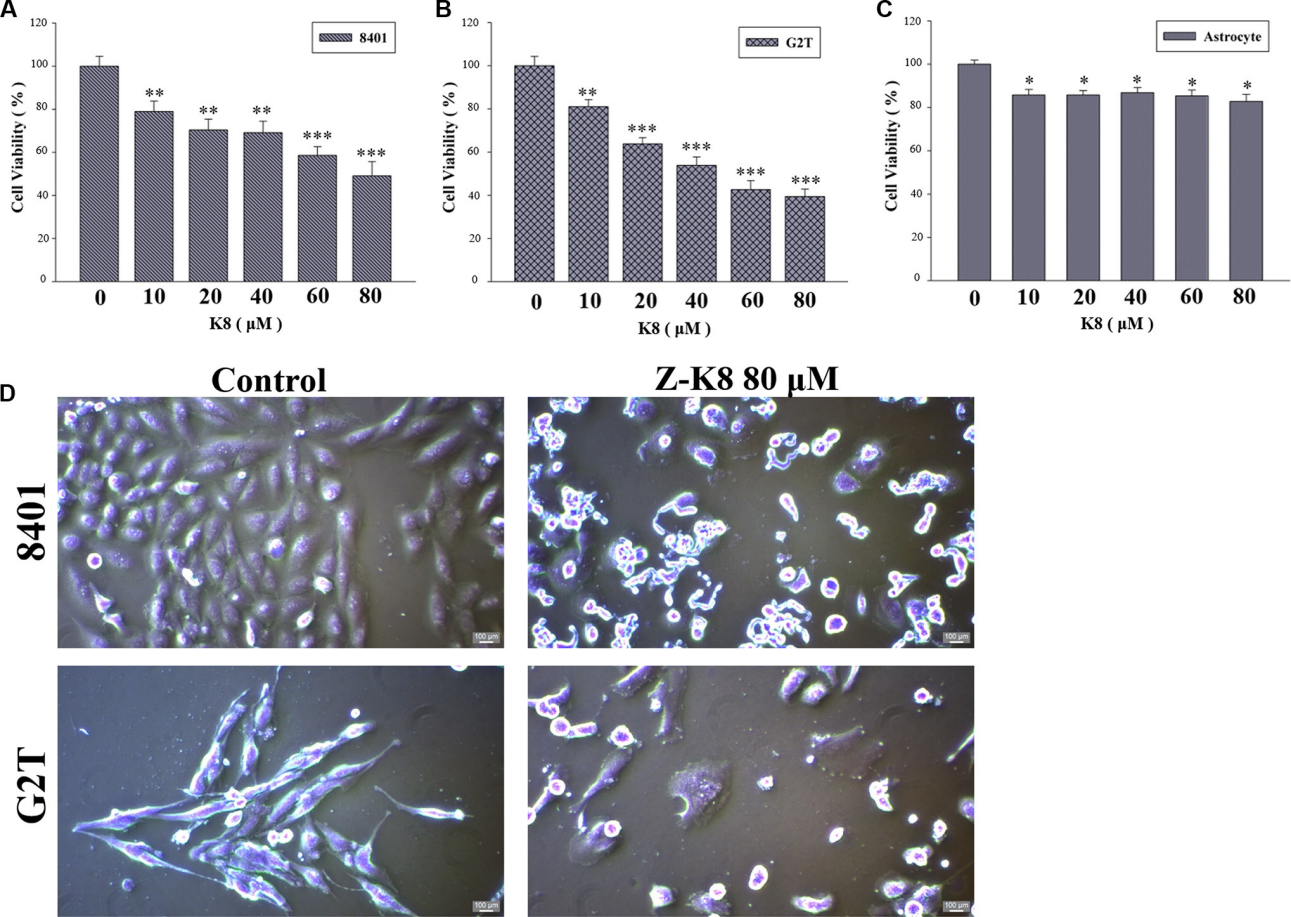
K8 induced cell death in glioblastoma cell lines (**A**–**B**) Glioblastoma cancer cell lines 8401 and G2T were treated with K8 for 24 hrs in a dose dependent manner. (**C**) Human primary astrocyte. Cell viability was detected by MTT assay. K8 reduced 8401 and G2T cell viability but does not affect astrocyte viability. Level of statistical significance: *p < 0.05, **p < 0.01, ***p < 0.001. (**D**) Effects of morphology and chromatin change of K8 in 8401 and G2T. Morphological examination of K8-induced giant cells after cells were treated with 80 μM of K8 for 24 hrs.

We tested the cytotoxicity of K8 on GBM cancer cells, 8401, G2T and astrocyte. MTT Assay tests revealed that K8 exerted a strong anti-proliferative effect on the GBM cell lines (Figure 2A–2C). Compared with untreated cells, K8-treated 8401 and G2T cells floated on the surface of the medium, which is a typical feature of cells undergoing apoptosis (Figure 2D).

### K8 causes glioblastoma cell cycle arrest and apoptosis

**Figure 3:**
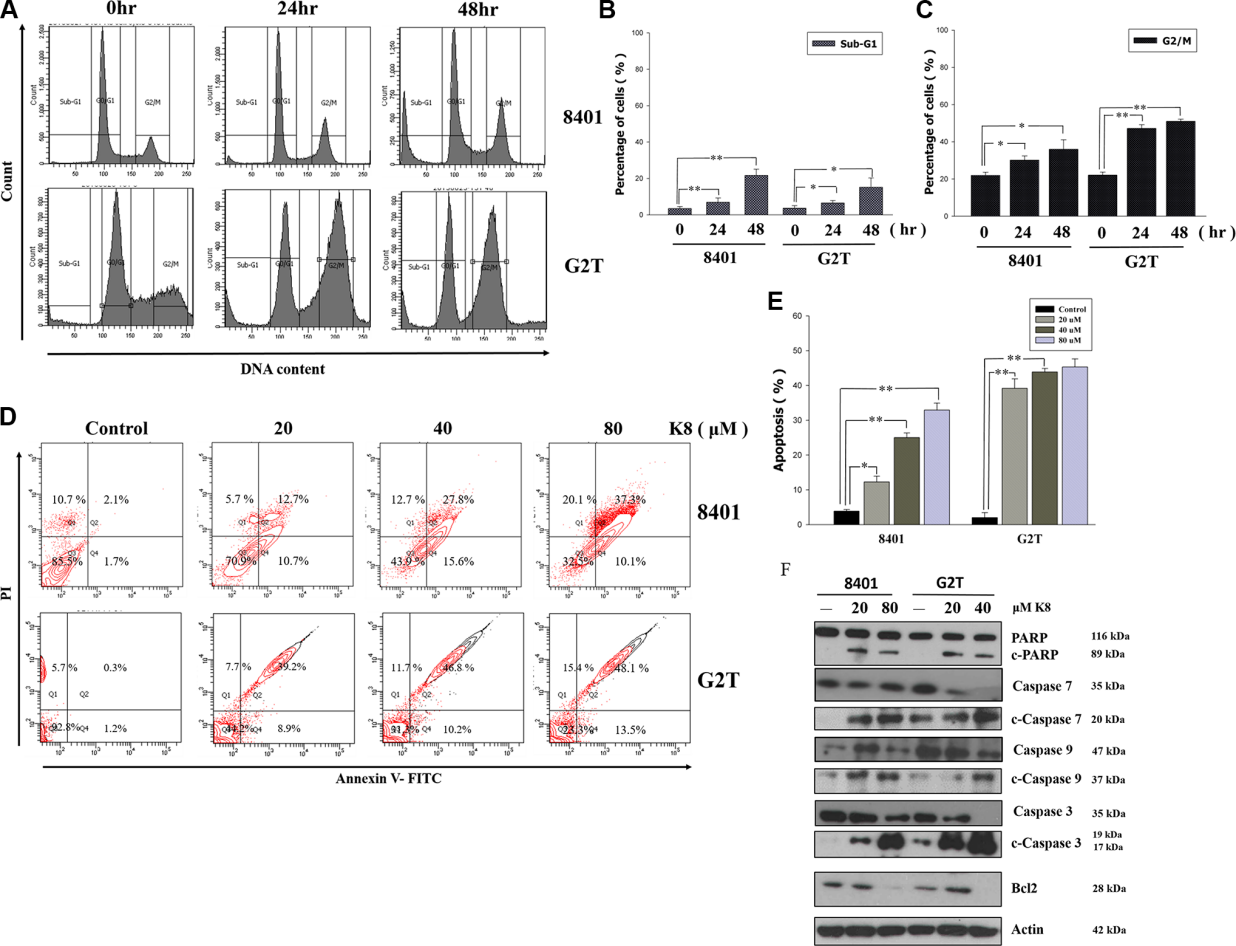
Effects of K8 on cell cycle and apoptosis in glioblastoma cell lines 8401 and G2T (**A**) The cells were treated with K8 80 uM for 0, 24, 48 hrs and analyzed for cell distribution by flow cytometry. (**B**–**C**) The subG1 and G2/M cell population were quantified. (**D**–**E**) 8401, G2T were treated with K8 20, 40, 80 uM for 24 hrs and then analyzed for AnnexinV-PI double stain to detect for cell apoptosis. (**F**) After K8 treatment of 8401 and G2T cell lines apoptosis-related protein was expressed. Experiments were carried out at least three times. Level of statistical significance: *P < 0.05, **p < 0.01.

To investigate the effects of K8 on cell cycle progression, GBM cell lines were exposed to IC50 of K8 for 0, 24 and 48 hours, and the cell cycle phase distribution was analyzed using FACS (Figure 3A). The quantification of untreated 8401 cells showed that 61.8% of the cells were in the G0/G1 phase, 14.5% were in the S phase, and 20.4% were in the G2/M phase of the cell cycle 48 hours after plating. The treatment of 8401 cells with 60 μM of K8 for 48 hours increased the percentage of cells in the G2/M phase to 26.6%, and reduced the amount of cells in G0/G1 and S phases to 44.1% and 15.7%, respectively. The subG1 population of cells exceeded 12.5%, and G2T cells treated with K8 60 μM for 48 hours, increased the percentage of cells in the G2/M phase from 22.1% to 49.6%. The subG-1 population of cells also exceeded 17.3%. These two cell lines went through cell cycle arrest in G2/M phase under K8 treatment (Figure 3B and 3C). At the same time, we also performed an AnnexinV-PI double stain to verify that 8401 and G2T cells treated with K8, depending on the dose, will undergo apoptosis. Two cell lines went through apoptosis in low dosages of K8 (8401 : 12.7%; G2T: 39.2%) and apoptotic population increase to 37.3% (8401) and 48.1% (G2T) under K8 80 μM treatment. (Figure 3D). The statistical result showed that K8 significantly caused these two cell lines to go through apoptosis (Figure 3E). Based on FACS data, we discovered that apoptosis played a role in K8-induced cell death. We found that K8-induced cell death was due to caspase pathway activation. After 8401 and G2T cells were treated with K8 for 48 hours, we harvested whole cell lysates for western blotting. PARP, caspase-3, caspase-9, and caspase-7 were upregulated and Bcl-2 was downregulated, following K8 treatment (Figure 3F). Thus, K8-induced cell death in 8401 and G2T cells in the caspase-dependent pathway.

### Knockdown *NAG-1* and *DDIT3* expression can restore 8401 cell viability

**Figure 4:**
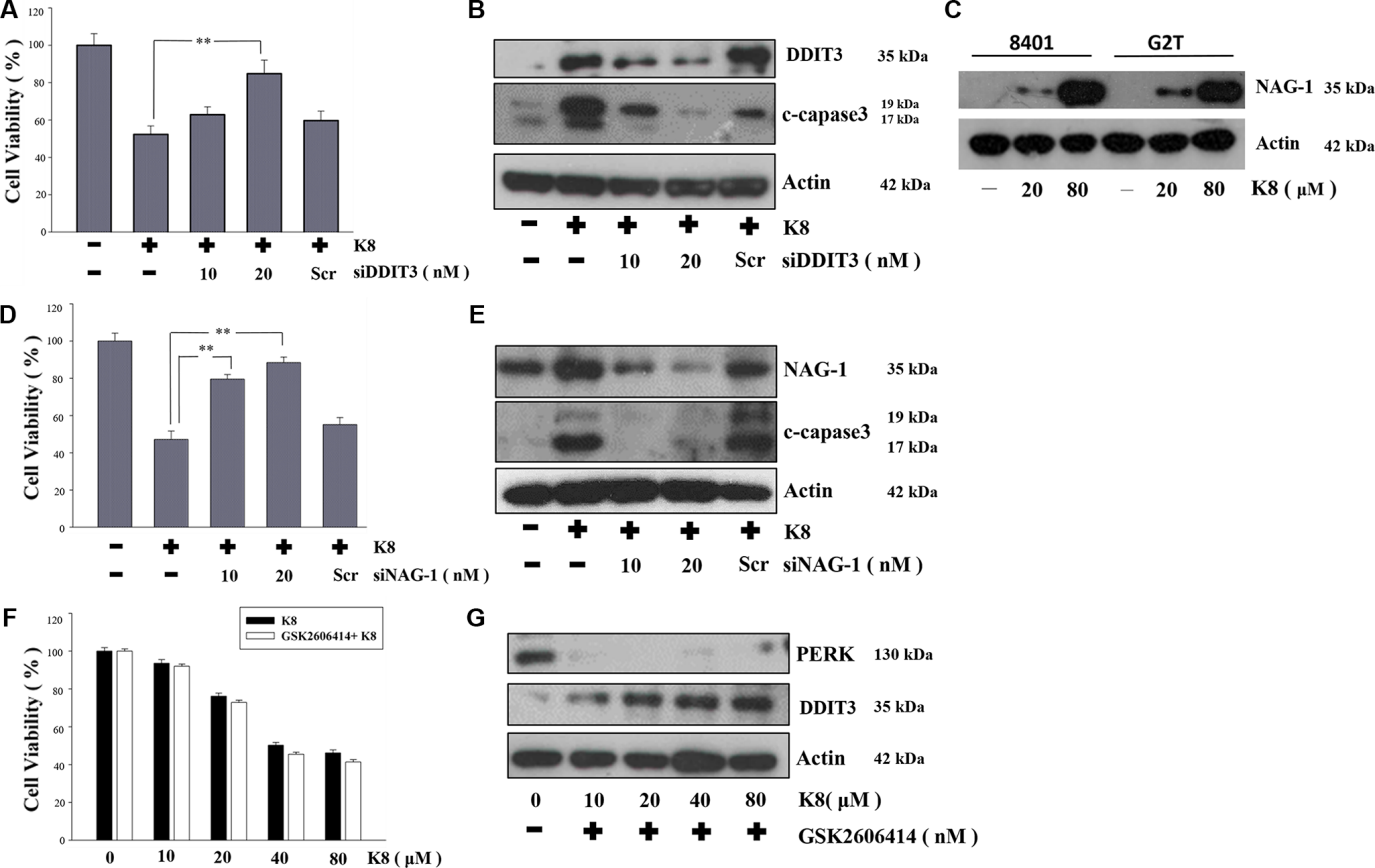
Effect of DDIT3, NAG-1, PERK on the induction of apoptosis by K8 in 8401 (**A**, **D**) 8401 cells were transfected with various concentrations of DDIT3 or NAG-1 siRNA (10, 20 nM ) and treated with K8 for 24 hrs. (**B**, **E**) Western blot analysis were performed for DDIT3, NAG-1 and cleaved capase3. (**C**) K8 induced NAG-1 expression in 8401 and G2T cell lines. (**F**) 8401 cells were first treated with 10 nM PERK inhibitor GSK2606414 for 24 hrs and then treated with K8 in a dose-dependent manner. (**G**) Western blot analysis was performed for PERK and DDIT3. Level of statistical significance: *p < 0.05, **p < 0.01.

The significantly increased *DDIT3* expression played a crucial role in the investigation of the mechanism responsible for the K8-induced glioblastoma cell apoptosis. We used *DDIT3* siRNA to knockdown *DDIT3* protein expression, which restored the 8401 cell viability (Figure 4A), and inhibited apoptosis-protein-cleaved-caspase-3 expression (Figure 4B). Previous studies showed that K8 can also activate NAG-1 expression in lung and prostate carcinoma, but the mechanism is still unclear [15, 16]. In this study, we showed K8 activated NAG-1 expression not only in lung and pancreatic carcinoma, but also in glioblastoma (Figure 4C). And, we used *NAG-1* siRNA to silence NAG-1 expression, which was induced by K8 treatment. In our result, K8-induced NAG-1 expression led to 8401 cell apoptosis (Figure 4D–4E). We found 8401 and G2T cell lines had high levels of PERK, with no DDIT3 expression. Previous research showed that the PERK inhibitor GSK2606414 caused the prion-infected cell to escape when overloaded under ER stress and then undergoes apoptosis [17]. Our hypothesis is that K8 will induce DDIT3 with no PERK activation. Therefore, we used GSK 2606414 to inhibit PERK expression and treated 8401 and G2T cells with K8. Our data showed that K8 decrease the cell viability of glioblastoma when used in tandem with GSK 2606414 (Figure 4F). The western blotting revealed that GSK 2606414 inhibited PERK expression; K8 with GSK 2606414 induced DDIT3 expression (Figure 4G).

### K8 induced DDIT3, which led to NAG1 expression, and finally, 8401 cell undergo apoptosis

**Figure 5:**
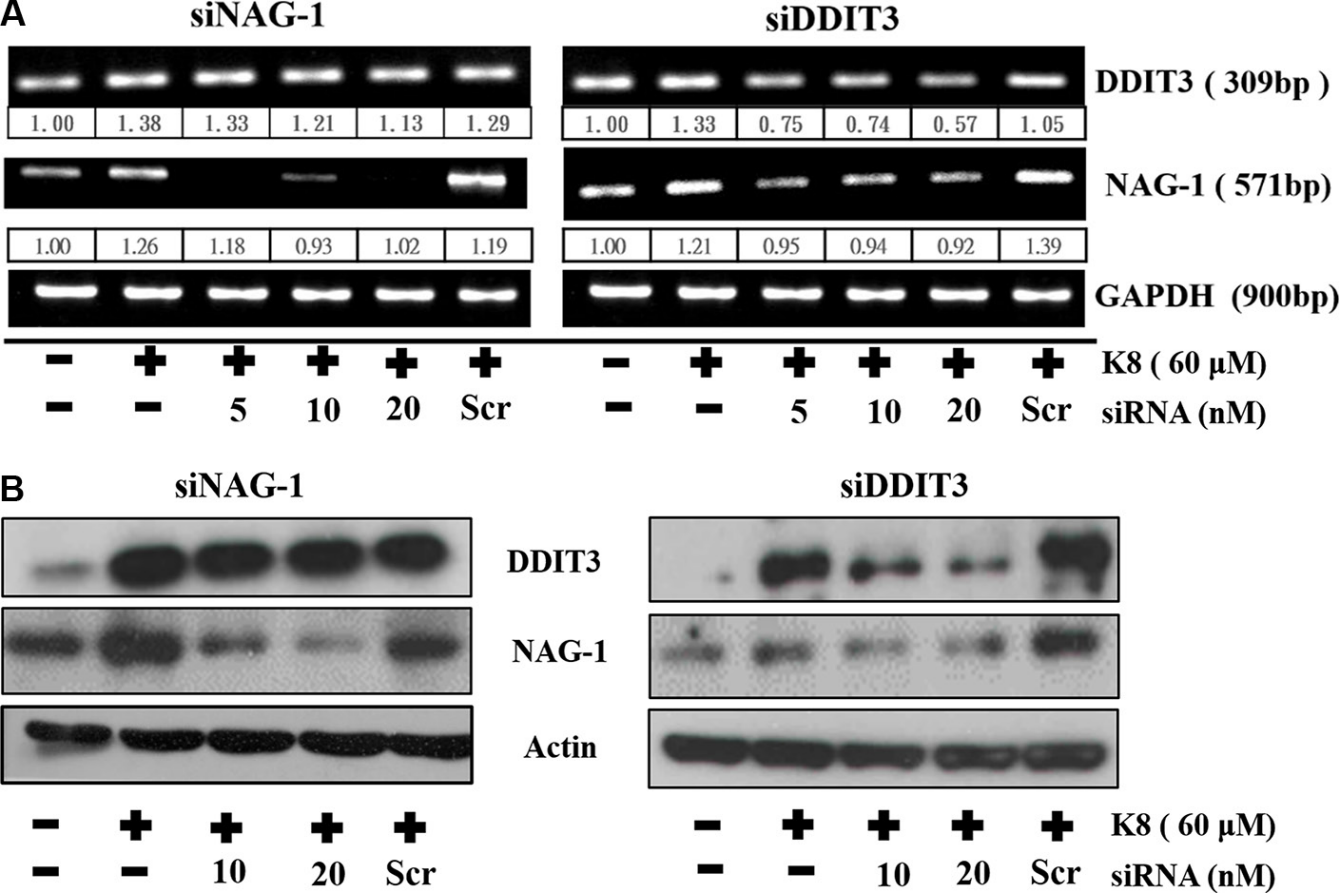
DDIT3 contributes to the regulation of NAG-1 gene expression (**A**) RT-PCR analysis of DDIT3 and NAG-1 expression in 8401 were treated with DDIT3 and NAG-1 siRNA. Cells were all transfected with siRNA which targeted DDIT3 or NAG-1 for 6 hrs prior to treatment with K8. (**B**) Western blot analysis of NAG-1 and DDIT3 in whole cell lysates of 8401 were harvested 24 hrs after treatment with DDIT3 or NAG-1 siRNA.

We discovered that the increase in *DDIT3* gene expression was accompanied by a considerable rise in *NAG-1* gene expression. *DDIT3* siRNA was used to inhibit *DDIT3* gene expression, which reduced *NAG-1* gene expression substantially, by more than 40%. Although we used siRNA transfection to knockdown NAG-1 expression, K8-induced DDIT3 expression remained unchanged in the transcription level (Figure 5A) and the translation level (Figure 5B) stages. Overall, these results indicate that DDIT3 regulates NAG-1 gene expression.

### K8 reduces glioblastoma tumor growth and increases tumor DDIT3 expression in a xenograft model

**Figure 6:**
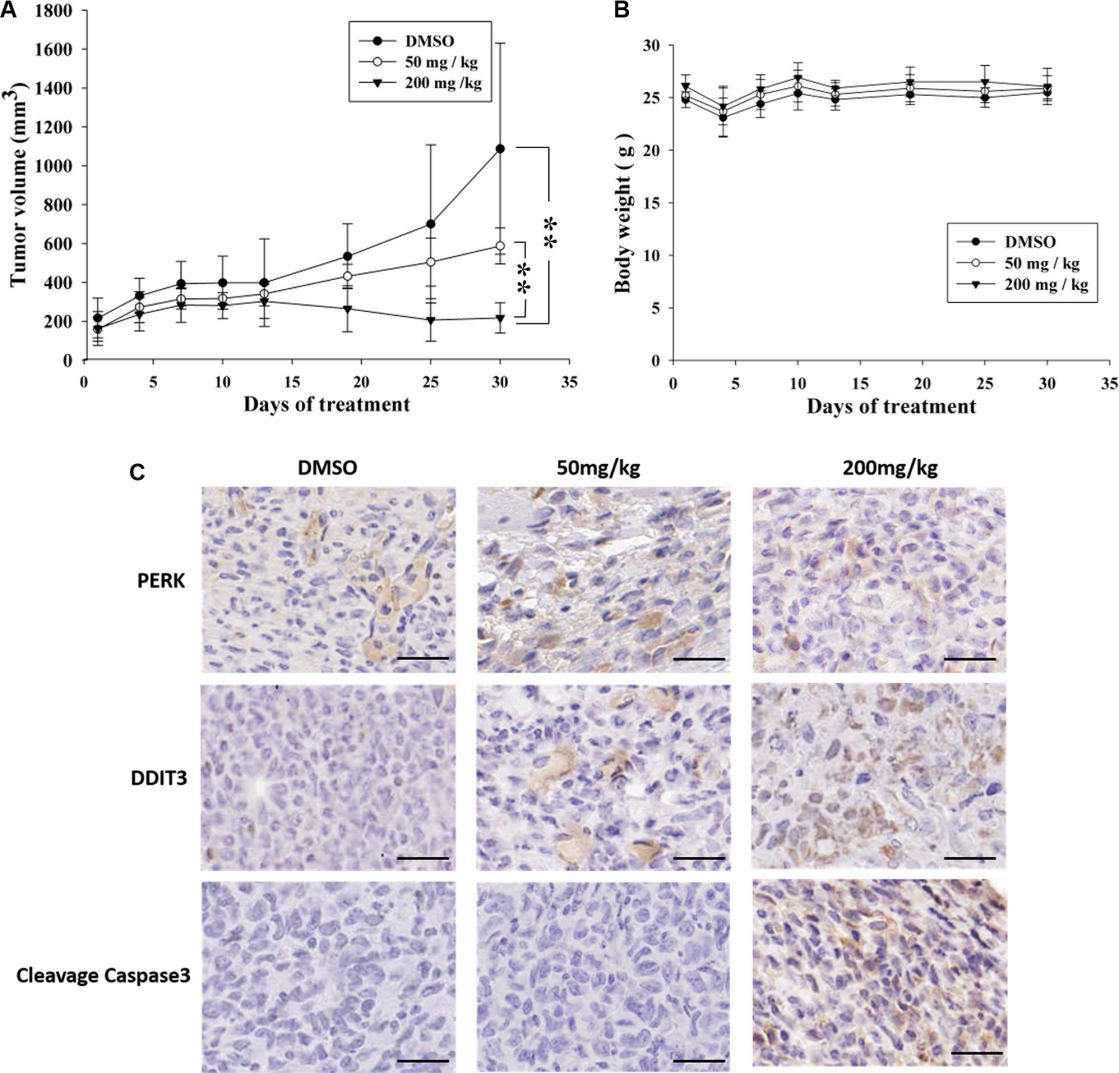
Antitumor effects of K8 in the nude mice xenograft model (**A**) Shown is the tumor growth curve with three different treatment groups: Control (DMSO), 50 mg/kg, and 200 mg/kg (each group *n* = 6). (**B**) Mean body weights for each group during the treatment period. (**C**) Immunohistochemistry images of DDIT3 and PERK and cleaved caspase3 are staining of tumor sections. Brown coloration indicates apoptotic cells (original magnification 200 ×). Scale bar is 20 μm.

**Figure 7:**
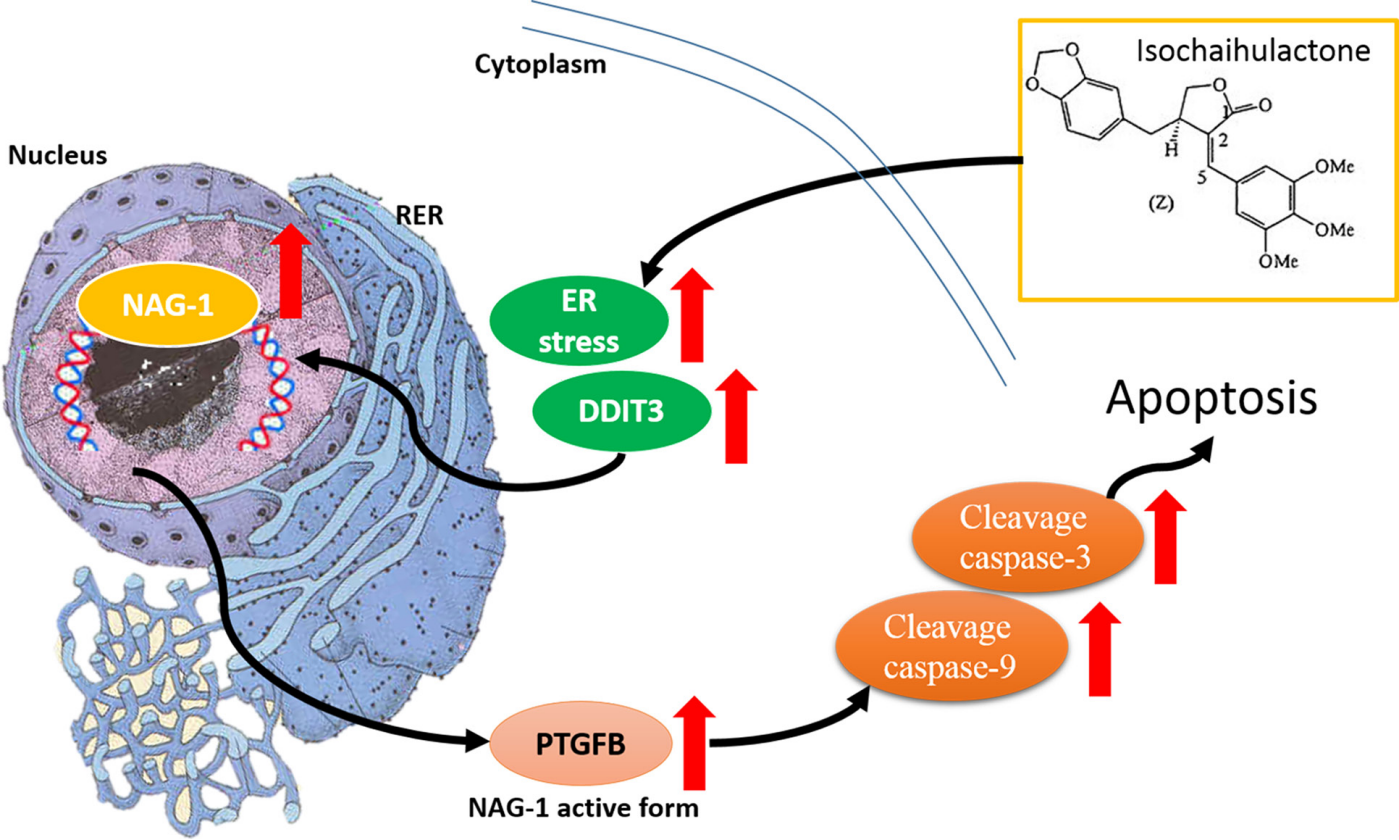
K8 induction of DDIT3 expression which leads to the modulation of NAG-1, and the whole process results in GBM cell apoptosis Scheme summarizes the K8-induced signaling pathway in glioblastoma cell apoptosis.

The xenograft model that was used to evaluate the *in vivo* antitumor activity of K8. Approximately 1 × 10^6^ 8401 cells was injected into the back of nude mice. After the tumor grew approximately 100 mm^3^ in volume, the mice were randomized into 3 groups: (1) Control (vehicle), (2) Low-dose treatment (50 mg/kg), and (3) High-dose treatment (200 mg/kg). Treatment groups (6 animals each) were given daily subcutaneous injections of K8 for 5 days. The result revealed that K8 inhibited tumor growth at low doses, whereas K8 induced tumor regression at high doses (Figure 6A); weight loss was not observed during the experiment (Figure 6B). Additional histological examination revealed DDIT3 expression *in vivo* (Figure 6C). The data showed that K8 did not cause PERK expression; however, the DDIT3 and cleaved caspase-3 was expressed with higher doses of K8. K8-induced apoptosis is mediated by the DDIT3-modulated NAG-1 apoptotic pathway (Figure 7).

## DISCUSSION

Increasing evidence supports the hypothesis that ER stress is very important in many diseases, including cancer [18]. We first analyzed the ER stress conditions in various GBM cell lines. We demonstrated that GBM cells experienced ER stress with increased levels of PERK, and that GRP78 was overexpressed in these cell lines. However, DDIT3 levels were low. Under K8 treatment, GRP78 was not affected and PERK was downregluated; however, DDIT3 was upregulated. Miyake et al. found that GRP78 overexpression prevented DDIT3 expression which also inhibited cell apoptosis. Tunicamycin, an ER stress inducer, can causes ER stress overload and prevents GRP78 from functioning, thus leading cells to undergo apoptosis [19]. Many researchers have developed anti-GRP 78 drug to block the ER stress repair system which induces cancer apoptosis. [13, 20]. The present study results revealed that DDIT3 and NAG-1 were upregulated, leading to tumor cell apoptosis with K8 treatment. Therefore, we found another pathway different from PERK–eIF2α axis, where K8 induces DDIT3 expression directly.

This study is the first to report that DDIT3 regulates NAG-1 expression with K8 treatment. According to our results, DDIT3 regulates NAG-1 expression at the transcriptional level, which means that DDIT3 may alter NAG-1 mRNA expression and its stability. The ER stress induced the DDIT3-mediated cytosolic translocation of HuR and the formation of stress granules. The HuR positively affected NAG-1 gene regulation by stabilizing NAG-1 mRNA during ER stress. The NAG-1 expression is modulated at various levels, including transcriptional and post-transcriptional regulation [21]. Rottlerin, a selective inhibitor of the novel isoforms of protein kinase C δ (PKC δ), significantly induced DDIT3 and NAG-1 expression in HT26 colon carcinoma, but DDIT3 siRNA did not affect rottlerin-induced NAG-1 expression [22].

Previous studies have shown that K8 caused growth inhibition by inducing the G2/M phase arrest of human lung adenocarcinoma and prostate cancer cells, and induced NAG-1 expression considerably [23, 24]. In the cytoskeleton, K8 inhibited tubulin polymerization and its pattern, but the molecular mechanism is not adequately understood [25]. The DDIT3-induced apoptosis via the autophagy pathway was reported in a previous study [26]. DDIT3 expression also affected cell cycle-related protein p21, cyclin-dependent kinase (CDK1), and CDK2 [27].

## MATERIALS AND METHODS

### Cell lines and chemical reagents

The glioblastoma cell lines 8401, 8901, U87, GBM-22 TMZ resistence cell line (G2T), were purchased from Bioresource Collection and Research Center (BCRC). RG2, GL261, were purchased from the American Type Culture Collection (ATCC). 131TXM and 1XM, two glioblastoma stem cell lines, were generous gifts from Dr. Dueng-Yuan Hueng. Roswell Park Memorial Institute (RPMI) 1640 medium, Dulbecco modified Eagle's medium (DMEM), FBS, penicillin, streptomycin, and trypsin–EDTA were purchased from Gibco. Dimethyl sulfoxide, MTT, and Lipofectamine 2000 were purchased from Invitrogen (Carlsbad, CA, USA). Caspase-3 antibody, anti-cleavage caspase-3 antibody, anti-cleavage caspase-7 antibody, anti-cleavage caspase-9 antibody, DDIT3 antibody, NAG-1 antibody, and monoclonal β-actin antibodies were purchased from Sigma–Aldrich Co. (St. Louis, MO, USA). PERK antibody and GRP78 antibody were purchased from Genetex. Fluorescein is thiocyanate, Annexin V Apoptosis Detection Kit I, RNA isolation kit, and Polyvinylidene difluoride membranes were purchased from BD Pharmingen.

### Cell culture

Human glioblastoma multiform cell lines were maintained in an RPMI 1640 medium containing 10% FBS and 100 ng/mL concentrations of both penicillin and streptomycin. These were maintained at 37°C in a humidified atmosphere with 5% CO_2_. Glioblastoma stem cell lines 131TXM and 1XM were then maintained in Eagle's minimum essential medium with 10% FBS and 100 ng/mL concentrations of both penicillin and streptomycin at 37°C in a humidified atmosphere with 5% CO_2_. All cultures were free of mycoplasma.

### Cell viability assay

Cells were seeded into 96 well plates at 5,000 cells per well, which were incubated overnight. After incubation for 24 hours, cells were treated with K8 at 0, 10, 20, 40, 60, 80 μM concentrations. Following treatment for 24 hours, we added new medium, containing 5 mg/ml 3-(4,5-cimethylthiazol-2-yl)-2,5-diphenyl (MTT), which was incubated for 1 hour. We then withdrew the medium and added 100 μL of Dimethyl sulfoxide to each well. Absorbance was measured at 595 nm on a spectrophotometer. All experiments were performed in triplicate.

### Cell cycle assay

Cell cycle progression was assessed via flow cytometry analysis, following DNA staining to quantify the total amount of DNA. Approximately 5 × 10^5^ of 8401 and G2T cells were incubated with 60 μM K8 for the indicated time. The cells were harvested with trypsin–EDTA, collected, washed with PBS, fixed overnight with cold 75% ethanol, and then stained for 1 hour in the dark with a solution containing 45 mg/mL of Propidium iodide (PI), 10 mg/mL of RNase A, and 0.1% Triton X-100. The cells were then passed through a FACScan flow cytometer, equipped with a 488 nm argon laser, to measure the DNA content. Cell Quest 3.0.1 (Becton Dickinson; Franklin Lakes, NJ, USA) and ModFitLT 2.0 software were used in order to obtain and analyze data.

### Annexin V and PI double-staining assay

Apoptosis was analyzed by using the method by van Engeland et al. (1998) to detect the integrity of the cellular membrane and the externalization of phosphatidylserine. Approximately 10^6^ cells were grown in 35-mm-diameter plates. The cells were treated with various herbal extracts and chemicals according to the experimental design, and were then labeled with 10 μg/mL of annexinV-FLOUS and 20 μg/mL of PI before harvesting. After labeling, the cells were washed with a binding buffer and harvested via scraping. They were resuspended in a binding buffer at a concentration of 2 × 10^5^ cells/mL prior to flow cytometry analysis (FACScan). WinMDI 2.8 software was used to analyze data. The percentage of cells undergoing apoptosis was determined through 3 independent experiments.

### RNA extraction, reverse transcription polymerase chain reaction, and quantitative polymerase chain reaction

For the microarray and reverse transcription polymerase chain reaction (RT-PCR) experiments, human GBM8401 cells were treated with 60 μM K8 for 24 hours. RNA was extracted with TRIzol (Invitrogen) and then purified using RNeasy (Qiagen) in accordance with the manufacturer's instructions. Total RNA from the GBM8401 cells was isolated as mentioned. The cDNA was synthesized through the reverse transcription of 2 μg of total RNA by using oligo(dT) and SuperScript II RNA reverse transcriptase (Invitrogen). The cDNA was then used as a template to amplify the corresponding DNA fragments through PCR, utilizing 2 sets of synthetic oligonucleotide primers. DNA amplification was performed using PCR with a Thermocycler 2400 (PerkinElmer Life and Analytical Sciences, Boston, MA, USA) under the following conditions: 30 cycles of denaturation at 95°C for 45 seconds, annealing at 58°C for 45 seconds, and extension at 72°C for 15 seconds. The primers used for PCR amplification are listed in Table 2. The PCR products were separated on 2% agarose gels, stained with ethidium bromide, and visualized using the FluorChem imaging system (Alpha InnoTech, San Leandro, CA, USA). The levels of glyceraldehyde-3-phosphate dehydrogenase were used as the control.

### Protein extraction and western blotting

Approximately 2 × 10^6^ cells were cultured in two 100-mm dishes, and then incubated with various concentrations of K8 for 24 hours. The cells were lysed on ice with 100 μL of a lysis buffer 50mM Tris-HCl, 0.5 M NaCl at a pH of 7.5, 5 mM MgCl_2_, 0.5% Nonidet P-40, 1 mM phenylmethylsulfonyl fluoride, 1 μg/mL pepstatin, and 50 mg/mL leupeptin. The cells were then centrifuged at 14,000 g for 20 minutes at 4°C. Protein concentrations in supernatants were quantified using a bovine serum albumin protein assay kit. Electrophoresis was performed on a NuPAGE Bis-Tris Electrophoresis System using 50 μg of reduced extract per lane. Resolved proteins were then transferred to polyvinylidene difluoride membranes, which were blocked with 5% nonfat milk for 1 hour and probed overnight with an appropriate dilution of primary antibodies at 4°C. The membranes were washed 3 times with 0.1% Tween 20 and incubated with horseradish peroxidase-conjugated secondary antibody at a 1:5000 dilution for 1 hour at room temperature. And we use β-actin as loading control. The ECL detection system was used in accordance with the manufacturer's instructions.

### RNAi transfection

The DDIT3 siRNA targeting the sequence AGA-GGC-AUA-CCA-AGA-UCC-A and NAG-1 siRNA targeting the sequence GAC-UCC-AGA-UUC-CGA-GAG-U were synthesized (Ambion, Austin, TX, USA). Cells at 50–60% confluence were transfected with the transfection reagent lipofectamine 2000 in accordance with the manufacturer's protocol, with DDIT3 or NAG-1 siRNA (Ambion) at concentrations of 5–20 nM for 48 hours. The transfected medium was removed, and the cells were treated with K8 or a vehicle for up to 48 hours. After incubation, RNA was isolated for RT-PCR, and the cells were collected for apoptosis analysis.

### Tumor xenograft model

Xenograft mice were used as a model system to examine the *in vivo* cytotoxic effect of K8, and they were injected subcutaneously with 8401 cells (2 × 10^6^ cells/mL). BALB/c Nude (nu/nu) mice were purchased from the National Science Council (Taipei, Taiwan), and all procedures were performed in compliance with the standard operating procedures of the Laboratory Animal Center of Tzu Chi University (Hualien, Taiwan). Animal were housed with an inverse 12 hours day-night cycle with lights on at 8:30pm in a temperature(22 ± 1°C) and humidity (55 ± 5%) controlled room. Prior to surgery the animal were housed to familiar new environment for 7 days. Experiments were performed using 6–8 week-old mice weighing 23–27 g. When the tumor volume reached 240–260 mm^3^, the animals were separated randomly into 3 groups: control, low-dose (50 mg/kg), and high-dose (200 mg/kg). Each group consisted of 6 mice at Day 0. Daily subcutaneous administrations of K8, dissolved in DMSO, were performed from Days 0–4 away from the inoculated tumor sites. The control group was treated with DMSO only. The mice were weighed 3 times per week, up to Day 30, to simultaneously monitor the effects and tumor volume by measuring the length (L) and width (W) of the tumors. The tumor volume at Day *n* (TVn) was calculated as TV (mm^3^) = (L × W^2^/2). The relative tumor volume at Day *n* (RTVn) versus that at Day 0 was expressed as follows: RTVn = TVn/TV0. Tumor regression (T/C, in %) in the treated versus control mice was calculated as follows: T/C (%) = (mean RTV of treated group)/(mean RTV of control group) × 100. Xenograft tumors, as well as the vital organs of the treated and control mice, were harvested and fixed in 4% formalin, embedded in paraffin, and cut into 4-mm sections for histological examination.

### Immunoblotting

Each group of subcutaneous GBM 8401 tumors were fixed in 4% formalin at 4°C for 16 hours and then embedded in paraffin. Paraffin sections were deparaffinized with xylene and rehydrated ethanol solutions graded from 75–95%. Then the sections were incubated with a blocking solution, which contained hydrogen peroxide and protein blocks for 20 min at room temperature, followed by incubation overnight with anti-DDIT3 rabbit polyclonal antibody in the blocking solution at 4°C. After that, we used the UltraVision Quanto Detection System (Thermo Scientific, Waltham, MA, USA) to detect the signal. Finally, the sections were counterstained with hematoxylin, mounted, scanned by Aperio Digital Pathology Slide Scanners (Leica Biosystems, CS2) at a magnification of 200, and photographed.

### Statistics

Immunoblot bands and gel bands were being quantitative by ImageJ software (Version 1.6). Statistical analyses were performed with Microsoft Excel 2016. Targeted bands were normalized with β-actin and GAPDH quantitative value. Data are shown as the mean ± SD. Statistical differences were analyzed using the Student *t* test for normally distributed values, and *P* values < 0.05 were considered significant. For multiple comparisons, one-way ANOVA was performed, followed by Tukey's range test, and a statistically significant difference was considered when *P* < 0.05.
